# The Warping of Plates

**Published:** 1862-01

**Authors:** J. H. Hyde

**Affiliations:** Lewistown, Ill.


### THE WARPING OF PLATES.

BY DR. J. H. HYDE, OF LEWISTOWN, ILL.

Much has been said, at various times, on the warping or springing of plates, during the process of soldering; but no one, as far as I know, claims to have discovered any means that prevents this difficulty in all cases, hence I will venture—would have ventured sooner but for diffidence—to give a brief statement of a plan I adopted about three years ago, which has not failed in any case to meet my wishes entirely. Indeed, I feel the same confidence in the identity of the fit after soldering that I do in my ability to solder the teeth to the plate. But, to the plan:—

Take, for example, a full upper set. After arranging the teeth as usual, preparatory to soldering, turn the points of them downward, on a piece of paper on the table, and before investing, take a piece of wire, as large as can be readily bent with the pliers, and long enough to reach from one end of the alveolar groove to the other, and bend it so as to fit the groove. When bent nearly to the proper position, it can be readily adapted by swaging in the dies. Lay this wire in the groove, and cut and fit four or five other pieces, (which may be smaller, if desired,) so as to reach across the plate; when bent to nearly fit it, and these cross-wires are to be laid at about equal distances from each other, from front to heel of the plate, with their ends resting inside of the curved wire in the groove. When the wires are thus adjusted, mix about equal portions of plaster and fine sand wet enough to run freely, and cover the wires with this, to the depth of a quarter of an inch (more or less, if circumstances require.) When the plaster is sufficiently firm to hold the wires in place, turn the piece over, back the teeth and finish in the usual mode. I use only annealed wire; and by preserving the pieces they may be used again repeatedly.

If any should adopt this plan I hope it will prove as satisfactory to them as it has to me.

